# PRICE (Protection, Rest, Ice, Compression, Elevation) vs. PEACE and LOVE (Protection, Elevation, Avoid anti-inflammatories, Compression, Education and Load, Optimism, Vascularization, Exercise) in adolescent lateral ankle sprain rehabilitation: a randomized prospective comparative study of muscle strength and dynamic balance

**DOI:** 10.1186/s13102-026-01651-7

**Published:** 2026-03-17

**Authors:** Matas Meškauskas, Saulius Rutkauskas, Miglė Misiūnienė, Juozas Žumbakys, Jūratė Tomkevičiūtė, Dalius Malcius, Emilis Čekanauskas

**Affiliations:** 1 Department of Pediatric Surgery, Lithuanian University of Health Sciences, Kaunas, LT-44307 Lithuania; 2 Department of Radiology, Lithuanian University of Health Sciences, Kaunas, LT-44307 Lithuania; 3 Department of Sports Medicine, Lithuanian University of Health Sciences, Kaunas, LT-44307 Lithuania; 4 Medical academy, Lithuanian University of Health Sciences, Kaunas, LT-44307 Lithuania; 5 Department of Physics, Mathematics and Biophysics, Lithuanian University of Health Sciences, Kaunas, LT-44307 Lithuania

**Keywords:** Lateral ankle sprain, PEACE and LOVE, PRICE, Y balance test, Biodex, Ankle instability, Acute lateral ankle ligament injury

## Abstract

**Background/Objectives:**

LAS is common in adolescents, yet early management strategies differ. The traditional PRICE + NSAIDs protocol focuses on short-term symptom relief, whereas the PEACE and LOVE framework emphasizes education, early optimal loading, and progressive exercise. This study compared functional recovery between these two approaches.

**Methods:**

A prospective randomized study enrolled 76 adolescents (12–17 years) with first-time LAS, allocated to PRICE + NSAIDs or PEACE and LOVE using a computer-generated randomization sequence with concealed envelope allocation; 65 completed follow-up (PRICE n = 32; PEACE and LOVE n = 33). Functional performance was assessed at 1–2, 5–7, and 12–15 weeks using Biodex isokinetic dynamometry and the Y-Balance Test composite score (YBT-CS). Outcomes were expressed as side-to-side deficits (uninjured − injured limb) and analyzed using two-way mixed repeated-measures analysis of variance, with Group (PRICE + NSAIDs vs. PEACE and LOVE) as the between-subject factor and Time (1–2, 5–7, and 12–15 weeks) as the within-subject factor.

**Results:**

Significant main effects of time were observed for IN strength at 60°/s (F = 5.73, p = 0.006) and 120°/s (F = 10.15, p < 0.001), EV strength at 120°/s (F = 6.82, p = 0.003), ankle ROM at 60°/s (F = 12.79, p < 0.001) and 120°/s (F = 13.09, p < 0.001), and YBT CS (F = 6.91, p = 0.002), indicating progressive recovery across follow-up. No significant group effects or time × group interactions were detected for any outcome (all p > 0.05).

**Conclusions:**

Both rehabilitation protocols were associated with progressive functional recovery following adolescent LAS. No statistically significant between-group differences or differential recovery trajectories were detected over 12–15 weeks. These findings suggest that an active, education-focused rehabilitation approach yields functional outcomes comparable to traditional PRICE + NSAIDs management in the short term. Larger studies with longer follow-up are required to determine whether clinically meaningful differences exist between protocols.

**Trial registration:**

ClinicalTrials.gov: NCT07287020; registered 3 December 2025 (retrospectively registered).

**Supplementary Information:**

The online version contains supplementary material available at 10.1186/s13102-026-01651-7.

## Introduction

### Comparison of PRICE and PEACE and LOVE protocols in adolescent lateral ankle sprain rehabilitation

Lateral ankle sprain (LAS) is one of the most frequent musculoskeletal injuries in active adolescents, yet wide variability persists in diagnostic methods and treatment pathways [1, 2]. Although LAS is often considered a minor injury, it can often lead to chronic ankle instability (CAI) which is characterized by recurrent sprains with ongoing pain, swelling, and/or “giving way” for more than 12 months after the primary injury, leading to physical activity restrictions and functional limitations [3, 4]. These long-term consequences emphasize the need for accurate diagnosis, evidence-based early management, and effective rehabilitation to preserve joint function and prevent re-injury [1–4]. Conservative treatment is recommended as the first-line approach for LAS, reserving surgery for persistent mechanical or functional instability after the failure of ≥ 6 months active rehabilitation [1, 4]. For more than 20 years, acute care has relied on the PRICE (Protection, Rest, Ice, Compression, Elevation) approach, combined with non-steroidal anti-inflammatory drugs (NSAIDs). However, guideline authors note a lack of high-quality evidence supporting PRICE as a universal strategy and warn that excessive rest and prolonged NSAID use may impair optimal tissue repair [1]. On the contrary, contemporary rehabilitation enhances early loading, progressive exercise, and patient education. A 2019 systematic review and meta-analysis reported that exercise-based rehabilitation reduces recurrent sprains risk compared with usual care, though optimal content and dosage remain unclear [5]. In order to avoid drawbacks of traditional treatment algorithm, PEACE and LOVE was suggested as a two-phase protocol covering immediate care (Protection, Elevation, Avoid anti-inflammatories, Compression, Education) and subacute recovery (Load, Optimism, Vascularization, Exercise), paying attention to education, early optimal loading, and graded activity [6]. Guidelines from the International Ankle Consortium (ROAST) highlight the importance of early clinical assessment, identification of sensorimotor deficits, and implementation of exercise-based rehabilitation strategies to minimize the risk of CAI [2]. Pediatric and adolescent studies published in 2019 also suggest age-specific considerations and exercise-based treatment tactics to improve long-term outcomes [4].

Recent evidence also questions routine use of anti-inflammatory modalities and prolonged cryotherapy. While NSAIDs and icing can provide short-term analgesia, both may interfere with natural inflammatory and vascular processes important for tissue healing [1, 7–9]. Thus, moderation rather than elimination of inflammatory response is recommended to facilitate optimal regeneration.

Traditional PRICE + NSAIDs and the PEACE and LOVE frameworks differ not only in clinical management but also in underlying physiological basis. PRICE concentrates primarily on early symptom control by reducing pain and swelling; however, prolonged rest and routine use of anti-inflammatory strategies may impair beneficial inflammatory cascades, macrophage activation, angiogenesis, and collagen remodeling required for optimal tissue healing [1, 7–9]. Conversely, PEACE and LOVE supports early, gradual mechanical loading and patient education to stimulate mechanotransduction, maintain neuromuscular function, and support collagen fiber alignment. This protocol aims to optimize tissue regeneration and functional recovery rather than simply controlling symptoms [6]. In this context, PEACE and LOVE represents a more biologically active and rehabilitation-driven strategy, whereas PRICE prioritizes short-term analgesia and acute symptom relief.

Despite these insights, direct functional comparisons between the traditional PRICE + NSAIDs protocol and the modern PEACE and LOVE approach in adolescent populations are limited. The objective of this study was to compare functional recovery following first-time LAS in adolescents treated with either the PRICE + NSAIDs protocol or the PEACE and LOVE framework, using objective measures of isokinetic ankle strength, range of motion (ROM), and dynamic balance over a 12–15-week follow-up period. By focusing on objective neuromuscular outcomes rather than return-to-sport time, this study aims to clarify which rehabilitation approach more effectively restores ankle stability and sensorimotor function in adolescents with LAS. We hypothesized that the PEACE and LOVE protocol would lead to better functional recovery compared to PRICE + NSAIDs at 12–15 weeks post-injury.

## Materials and methods

### Study design and participants

A prospective randomized study was conducted between February 2022 and January 2025 at the Lithuanian University of Health Sciences (LUHS) Kaunas Clinics, Department of Pediatric Orthopedics. A total of 76 patients (42 males and 34 females), aged 12–17 years, who presented to the LUHS Pediatric Emergency Department (ED) with a first-time lateral ankle sprain (LAS), were included in the study. Despite various inconveniences, 65 participants completed all stages of the study.

Inclusion criteria were: (1) first-time LAS, (2) acute injury phase of 1–4 days after trauma, and (3) absence of chronic ankle pain or fracture, except for minor avulsion fractures. Patients with previous ankle surgery, systemic diseases or neurological disorders were excluded. The physical examination of LAS was performed by a pediatric orthopedic surgeon. All ultrasound examinations were performed by a single musculoskeletal radiologist with over 15 years of experience (more than 50000 musculoskeletal ultrasound examinations) in musculoskeletal imaging to ensure diagnostic consistency. Diagnosis was confirmed using radiography and ultrasound imaging (MyLab 9 eXP 20071, Esaote S.p.A., CE 0123). LAS severity was classified according to the extent of injury to the lateral ankle ligament complex, which consists of the anterior talofibular ligament (ATFL), calcaneofibular ligament (CFL), and posterior talofibular ligament (PTFL). Injury grading was based on combined clinical examination and imaging findings (ultrasound and radiography), in accordance with established orthopedic descriptions [10].

Grade I LAS was defined as ligament stretching or microscopic injury without macroscopic fiber disruption, typically involving the ATFL only, with preserved ligament continuity on ultrasound and no mechanical instability on clinical examination. Grade II LAS was defined as a partial tear of the ATFL, with or without partial involvement of the CFL, demonstrated by ligament thickening, hypoechogenicity, or partial fiber discontinuity on ultrasound, accompanied by moderate pain, swelling, and functional limitation. Grade III LAS was defined as a complete rupture of the ATFL, usually with concomitant rupture of the CFL, and occasionally involving the PTFL. This category also included avulsion injuries, defined as detachment of the ligament from its bony insertion site with or without a small bone fragment, confirmed by ultrasound and/or radiography. Grade III injuries were associated with marked swelling, ecchymosis, and clinical signs of mechanical instability. Injuries involving the distal tibiofibular syndesmosis (high ankle sprains) were considered anatomically and biomechanically distinct and were excluded from this classification [11].

All participants received the same rehabilitation duration; outcomes focused on objective functional performance: (I) isokinetic muscle strength using the Biodex system and (II) proprioception/dynamic balance using the Y-Balance Test (YBT). The full study protocol and statistical analysis plan are available from the corresponding author on reasonable request. No important changes to the trial protocol or outcomes occurred after trial commencement. This study is reported in accordance with the CONSORT 2025 guidelines. 

### Ethical approval

Ethical approval for this study was obtained from the Kaunas Regional Biomedical Research Ethics Committee (Protocol No. 2022-BE10-0003). Prior to participation, written informed consent was obtained from all patients and their legal guardians prior to participation.

### Group allocation, randomization, blinding and treatment protocols

Depending on symptom severity, 30 patients (46.2%) were applied by temporary immobilization with a plaster cast in the ED. The initial outpatient appointment was planned to occur within 2–5 days following the injury event. At the first outpatient visit, casts were removed, and all patients were fitted with a lace-up ankle splint. Participants were randomly assigned to two treatment groups—PRICE + NSAIDs (Group A) or PEACE and LOVE (Group B)—using a computer-generated randomization sequence prepared by an independent researcher. Simple randomization was used without blocking or stratification. Each allocation code was sealed in an opaque, sequentially numbered envelope to maintain allocation concealment. This was a parallel-group randomized comparative study with 1:1 allocation and a superiority framework. At the first outpatient visit, the patient personally selected and opened the next envelope in sequence to reveal the assigned protocol. The enrolling clinician had no access to the random allocation sequence prior to envelope opening. Patients formed two to two age- and sex-homogeneous groups:

Group A (*n* = 32) — treated according to the traditional PRICE combined with NSAIDs (Ibuprofen prescribed according to body weight 3 times per day) (males 62.5%, females 37.5%). Group B (*n* = 33) — treated using the PEACE and LOVE approach (males 51.5%, females 48.5%).

Complete treatment protocols for both groups are provided and can be found in Appendix A, Table 1. To minimize bias, outcome assessors were blinded to treatment assignment. The musculoskeletal radiologist performing ultrasound diagnostics and the sports medicine physician conducting Biodex isokinetic and Y-Balance testing were both unaware of participants’ group allocation. Patients and the treating orthopedic surgeon were not blinded due to the practical nature of the treatment protocols. Orthopedic surgeon had to assign the corresponding treatment, such as prescribing NSAIDs, cold therapy, instructing exercises, which required knowledge of the allocation. This procedure ensured random sequence generation, maintained allocation concealment until the moment of treatment initiation, and preserved assessor blinding throughout diagnostic and functional evaluations. Adverse events, including medication-related side effects and re-injury, were monitored throughout follow-up via clinical visits and patient self-report. Patients and the public were not involved in the design, conduct, reporting, or dissemination of this research.

Both interventions were delivered by a pediatric orthopedic surgeon according to the assigned protocol. Adherence was generally good, with occasional non-compliance related to early return to sports or splint use. No major protocol deviations were observed. 

### Outcome measures

Functional recovery was assessed through objective biomechanical testing performed at 1–2 weeks, 5–7 weeks, and 12–15 weeks after injury. The first functional assessment was scheduled at 1–2 weeks post-injury to allow reduction of acute pain and swelling, as isokinetic testing during the immediate post-trauma period may be limited by pain and would not provide valid maximal strength measurements. Isokinetic muscle strength was evaluated using the Biodex Medical Systems 4 (model 835 − 210, CE 0413, USA; serial number: 09022707) dynamometer to measure ankle ROM and peak torque to body weight (Peak TQ/BW) during inversion (IN) and eversion (EV) movements. Testing of ankle EV (primarily the peroneus longus, peroneus brevis, and fibularis tertius) and IN (mainly the tibialis anterior and tibialis posterior) muscles, as well as ROM testing, was performed in collaboration with the LUHS Department of Sports Medicine. All examinations were performed by a single sports medicine physician to ensure diagnostic consistency. The Biodex isokinetic dynamometer was selected because dynamometric assessment of ankle IN and EV strength demonstrates good to excellent reliability, with intraclass correlation coefficients (ICC) reported approximately between 0.74 and 0.96 across studies [12]. To account for inter-individual variability in neuromuscular performance, all functional outcomes were expressed as side-to-side deficits, calculated as the difference between the uninjured and injured limbs (uninjured − injured). This within-subject approach allows each participant to serve as their own control and reduces potential misinterpretation that may occur when analyzing limb performance independently, particularly in adolescents whose absolute strength or balance values in the uninjured limb may be lower than population-based reference norms. A smaller side-to-side deficit was interpreted as improved functional symmetry and recovery.

Proprioception and dynamic balance were assessed using the YBT kit (Functional Movement Systems, Danville, VA, USA). The YBT was selected because it offers reliable (ICC 0.85–0.91), valid, and treatment-responsive assessment of dynamic balance and proprioceptive function after ankle sprain, making it appropriate to evaluate rehabilitation effectiveness in this population [13, 14]. Each participant performed a single-leg stance on the central platform while reaching the contralateral leg in three standardized directions—anterior, posterolateral, and posteromedial—using a validated YBT kit. The participant’s goal was to reach as far as possible in each direction while maintaining balance, ensuring heel contact with the platform and only light toe contact on the reach indicator. Each direction was tested three times per leg, and the maximum reach distance (cm) for each direction (anterior, posteromedial, posterolateral) was recorded. Limb length was measured from the anterior superior iliac spine to the distal tip of the medial malleolus. The YBT composite score (CS) was calculated by summing the maximal reach distances in all three directions, dividing this value by three times the limb length, and multiplying by 100, according to the standard YBT protocol. This normalized composite score enabled direct comparison between injured and uninjured limbs.

### Testing procedure

All participants were instructed to avoid physical activity for 24 h and abstain from food intake for at least 2 h prior to testing. Anthropometric measurements were collected before the procedure. A 5-minute warm-up on a stationary exercise bike was completed at an average of 60–70 rpm, 70 W intensity, and 110–130 bpm heart rate. The testing environment was standardized at 23 °C and 77% humidity. Following a 5-minute rest, participants were seated and secured with chest, waist, thigh, and calf straps on the Biodex system. Familiarization trials were performed at 25%, 50%, 75%, and 100% of the individual’s maximal effort. Each participant then performed three maximal trials at angular velocities of 60°/s and 120°/s, with 60 s of rest between repetitions. The uninjured limb was always tested first, followed by the injured limb. Verbal encouragement was provided to ensure maximal and consistent effort.

The YBT (Lower Quarter) was administered using a standardized protocol. All participants performed the test barefoot to eliminate footwear-related variability. The stance foot was placed flat on the center platform, and participants were instructed to keep their hands on the hips throughout testing. Prior to data collection, standardized verbal instructions and a demonstration were provided, followed by a familiarization phase. Three recorded trials were performed in each reach direction (anterior, posteromedial, posterolateral) for each limb, with standardized short rest periods between trials and directions to minimize fatigue. The testing order was consistent for all participants, and the uninjured limb was tested first. A trial was repeated if balance was lost, hands left the hips, the stance foot moved, or the reach foot provided support. Verbal encouragement was limited to standardized neutral cues to ensure consistency across participants [15].

### Statistical analysis

Data were analyzed using IBM SPSS Statistics (version 30.0; IBM Corp., Armonk, NY, USA). All outcomes were treated as continuous variables and expressed as side-to-side deficits (uninjured − injured). Continuous variables are presented as mean (SD) or median (IQR), depending on data distribution, and categorical variables are presented as frequency (%). Baseline group comparability for demographic and injury characteristics was examined using independent-samples *t* tests (continuous variables) and chi-square tests (categorical variables). To evaluate whether changes over time differed between the two rehabilitation approaches, each functional outcome (Peak TQ/BW in inversion and eversion, ROM at 60°/s and 120°/s, and YBT composite score) was analyzed using a two-way mixed repeated-measures ANOVA, with Group (PRICE + NSAIDs vs. PEACE and LOVE) as the between-subject factor and Time (T1: 1–2 weeks; T2: 5–7 weeks; T3: 12–15 weeks) as the within-subject factor. The primary inferential test for comparative effectiveness was the Group × Time interaction. When the interaction was statistically significant, follow-up analyses examined (i) between-group differences at each time point and (ii) within-group changes over time using planned pairwise comparisons based on estimated marginal means. Because these follow-up comparisons were considered hypothesis-driven and contingent on the omnibus interaction test, no Bonferroni adjustment was applied; exact *p* values are reported. When the interaction was not significant, results were interpreted primarily in terms of the main effects of Time and Group. Model assumptions were assessed by inspection of residuals. Homogeneity of variances between groups was evaluated using Levene’s test at each time point. Sphericity for the within-subject factor (Time) was assessed using Mauchly’s test; when violated, the Greenhouse–Geisser correction was applied. Effect sizes are reported as partial eta-squared (ηp²) for ANOVA effects, and 95% confidence intervals were used where available. All tests were two-sided, with statistical significance set at *p* < 0.05. Analyses were conducted per-protocol including participants who completed all three assessments; no imputation was performed for missing data.

### Power analysis and sample size determination

An a priori power analysis was conducted to estimate the minimum required sample size. Based on previous literature identifying IN strength as the most affected variable after LAS [16, 17], a mean side-to-side IN deficit was assumed. Earlier studies reported early IN strength deficits are most eminent in the first weeks post-injury [18]. Pilot data collected at the LUHS demonstrated a comparable early peak TQ/BW IN force deficit of injured leg, with a mean difference of 2.65% (with SD of 7.5%). The power analysis was performed in IBM SPSS Statistics (Paired-Sample Means procedure) assuming a Pearson correlation of 0.5, a two-tailed significance level of 0.05, and desired statistical single power value 0.8. Under these parameters, the minimum required sample size was calculated as 65 participants, which would be sufficient to detect a clinically meaningful change—assuming near-symmetrical strength restoration after 12–15 weeks of rehabilitation. Considering an anticipated attrition rate of approximately 15%, a total of 76 participants was determined to be necessary for study enrollment.

## Results

**Fig. 1:**
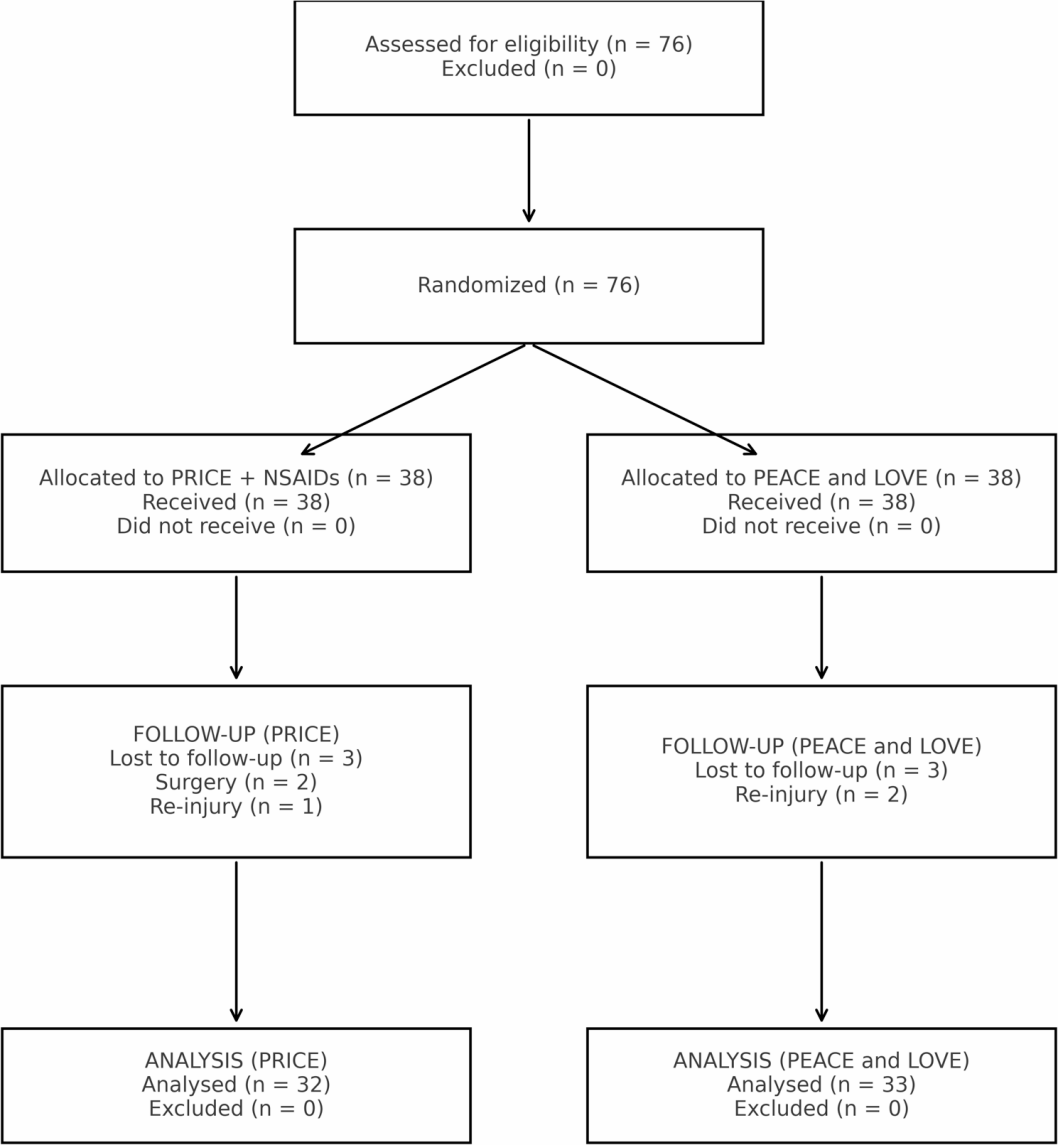
Flow chart of patient follow-up during 12-15 weeks of study

Unfortunately, not all participants were able to be followed up with for all 15 weeks, and 11 patients were lost due to various reasons. The flow chart that describes patient involvement in the study is presented in Fig. 1.

Analyses were conducted per-protocol and included participants who completed all follow-up assessments. No imputation was performed for missing data. A total of 65 patients (37 males [56.9%], 28 females [43.1%]) aged 12–17 years were included in the study. Sample characteristics are presented in Table 1. No serious adverse events or NSAID-related complications were reported.

**Table 1:** Sample characteristics of PRICE + NSAIDS and PEACE and LOVE groups

	PRICE + NSAIDS (*n* = 32)	PEACE and LOVE (*n* = 33)	Test statistic	*p*
Age in years (median (IQR))	14 (3)	14 (2.5)	|Z| = 0.806	0.420
Sex (male/female)	20/12	17/16	χ^2^ = 0.414	0.520
Height in cm (mean (SD))	175.3 (10.45)	174.4 (12.59)	|t| = 0.319	0.751
Weight in kg (mean (SD))	66.5 (11.15)	67.5 (17.73)	|t| = 0.278	0.782

|Z|: absolute standardized Mann-Whitney test statistic, χ^2^: Chi-square test statistic, |t|: absolute Student t test statistic

### Injury severity and initial management

Based on radiologic assessment and injury severity, 14 patients (21.5%) were diagnosed with Grade I (sprains of ATFL), 16 patients (24.6%) with Grade II (partial tears of ATFL or CFL) and 35 (53.8%) with Grade III (complete tear of ATFL, CFL or PTFL and/or avulsion of lateral malleolus or calcaneus) injuries (Table 2). Distribution of severity was similar between the two treatment groups (p > 0.05).

**Table 2:** Distribution of patients by injury severity in treatment groups

Severity	PRICE (*n* = 32)	PEACE and LOVE (*n* = 33)	Chi-square test statistic	*p*
I	7 (21.9%)	7 (21.2%)	0.013	0.993
II	8 (25.0%)	8 (24.2%)		
III	17 (53.1%)	18 (54.5%)		

Values are expressed as number (percentage) of patients. Severity classification was based on radiologic assessment: (I) Grade I (sprain of ATFL), (II) Grade II (partial tear of ATFL, CFL) and (III) Grade III (complete tear of ATFL, CFL or PTFL and/or avulsion of lateral malleolus or calcaneus)

**Fig. 2:**
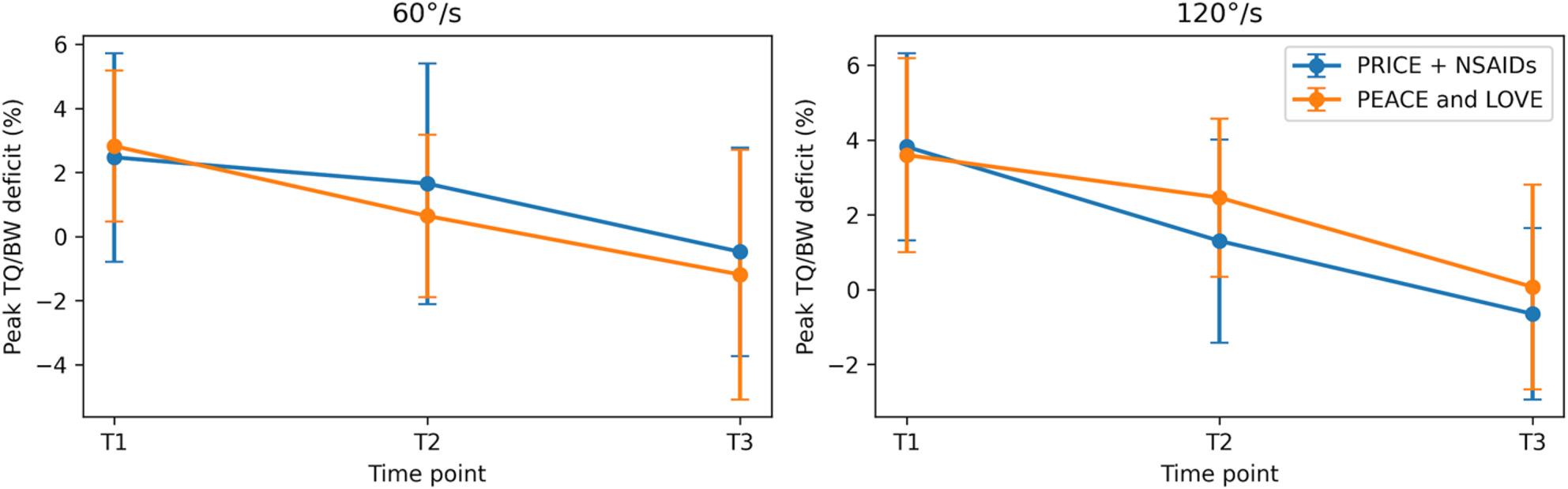
Inversion strength (Peak TQ/BW deficit, %) (Mean ±95%, CI)

**Fig. 3:**
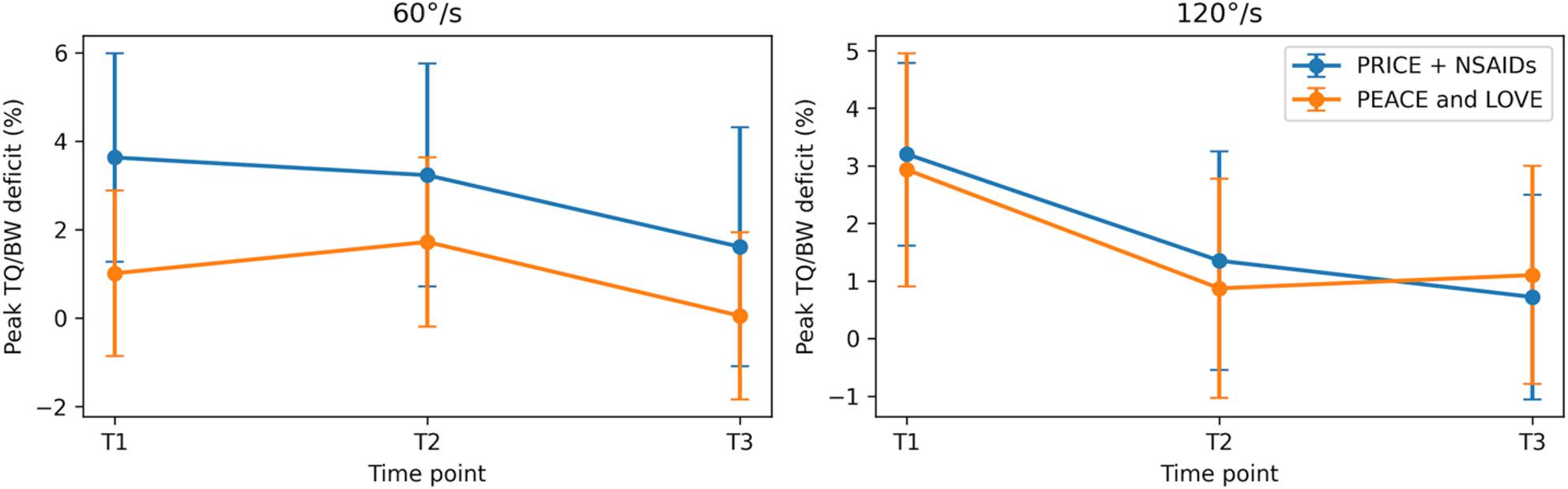
Eversion strength (Peak TQ/BW deficit, %) (Mean ±95%, CI)

**Fig. 4:**
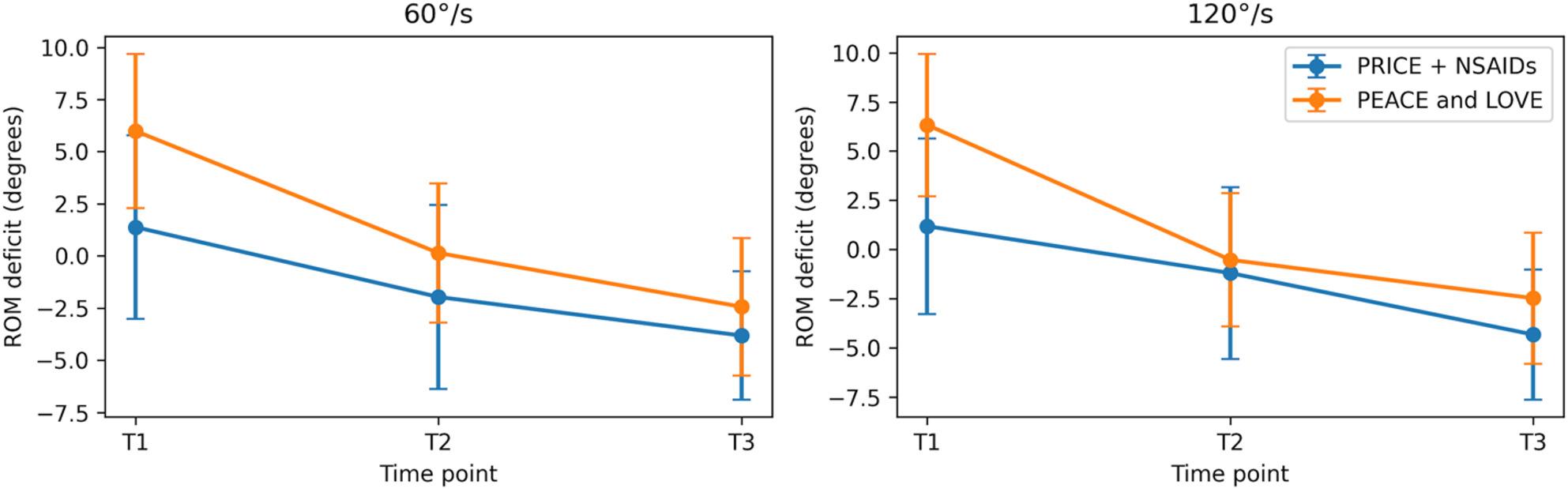
ROM deficit (degrees) (Mean ±95%, CI)

In the ED, temporary plaster cast immobilization was applied to 30 patients (46.2%). Notably, immobilization was frequently prescribed based on clinical symptoms rather than imaging confirmation, resulting in comparable application rates among both milder and more severe injuries (Table 3). Exploratory analyses examining the association between temporary plaster cast immobilization and functional outcomes were performed but were not part of the primary study objectives. These results are therefore presented in the Appendix B (Table 1 and Figures 1-4).

**Table 3:** Association between injury severity and application of plaster cast immobilization

Severity	Cast immobilization applied	No immobilization applied	Chi-square test statistic	*p*
I	6 (20.0%)	8 (22.9%)	0.935	0.627
II	6 (20.0%)	10 (28.6%)		
III	18 (60.0%)	17 (48.6%)		

Values are expressed as number (percentage) of patients. Cast immobilization was prescribed in the emergency department based primarily on clinical symptoms

### Comparison of Treatment Methods

Functional outcomes for the PRICE + NSAIDs and PEACE and LOVE groups are summarized in Table 4 . Mixed repeated-measures ANOVA revealed significant main effects of time for most strength, ROM, and dynamic balance outcomes, indicating progressive functional recovery across the follow-up period. No significant group effects or time × group interactions were observed for any outcome.

**Table 4:** Comparison of functional outcomes between treatment methods A (PRICE + NSAIDs) and B (PEACE and LOVE)

Variable	Time	Group		Time effect			Group effect			Time × Group		
		PRICE + NSAIDs	PEACE and LOVE	df	F	p	df	F	p	df	F	p
Peak TQ/BW IN (U–I) % at 60°/s	T1	2.47 (9.03)	2.82 (6.66)									
	T2	1.65 (10.42)	0.64 (7.16)	1.78, 111.85	5.73	0.006	1, 63	0.06	0.808	1.78, 111.85	0.24	0.759
	T3	-0.48 (9.02)	-1.19 (11.00)									
Peak TQ/BW EV (U–I) % at 60°/s	T1	3.63 (6.55)	1.01 (5.28)									
	T2	3.23 (6.99)	1.72 (5.39)	2.00, 124.36	3.01	0.053	1, 63	2.17	0.145	1.97, 124.36	0.35	0.700
	T3	1.61 (7.50)	0.05 (5.33)									
ROM (U–I) % at 60°/s	T1	1.38 (12.23)	5.99 (10.45)									
	T2	-1.97 (12.22)	0.14 (9.41)	2, 126	12.79	< 0.001	1, 63	1.74	0.192	2, 126	0.76	0.472
	T3	-3.82 (8.52)	-2.44 (9.28)									
Peak TQ/BW IN (U–I) % at 120°/s	T1	3.82 (6.96)	3.60 (7.33)									
	T2	1.30 (7.54)	2.46 (5.97)	1.62, 100.25	10.15	< 0.001	1, 62	0.15	0.699	1.62, 100.25	0.32	0.684
	T3	-0.65 (6.36)	0.07 (7.73)									
Peak TQ/BW EV (U–I) % at 120°/s	T1	3.20 (4.40)	2.93 (5.71)									
	T2	1.35 (5.26)	0.87 (5.36)	1.73, 106.94	6.82	0.003	1, 62	0.01	0.909	1.73, 106.94	0.24	0.752
	T3	0.72 (4.93)	1.10 (5.34)									
ROM (U–I) % at 120°/s	T1	1.18 (12.39)	6.33 (10.21)									
	T2	-1.20 (12.12)	-0.53 (9.54)	1.87, 115.92	13.09	< 0.001	1, 62	1.54	0.220	1.87, 115.92	1.34	0.266
	T3	-4.33 (9.17)	-2.48 (9.38)									
YBT CS (U–I) %	T1	2.72 (5.53)	2.60 (4.20)									
	T2	1.22 (6.43)	0.15 (2.80)	1.80, 113.52	6.91	0.002	1, 63	0.73	0.398	1.80, 113.52	0.33	0.698
	T3	1.03 (3.54)	0.09 (3.20)									

Values are presented as mean (SD). Outcomes are expressed as side-to-side deficits calculated as the difference between the uninjured and injured limb (uninjured − injured). Functional performance was assessed at three time points: T1 (1–2 weeks post-injury), T2 (5–7 weeks), and T3 (12–15 weeks). Isokinetic testing was performed at angular velocities of 60°/s and 120°/s for inversion (IN) and eversion (EV) strength and range of motion (ROM). Dynamic balance was assessed using the Y-Balance Test composite score (YBT-CS). Statistical analysis was conducted using mixed repeated-measures analysis of variance (ANOVA) with Time (T1–T3) as the within-subject factor and Group (PRICE + NSAIDs vs. PEACE and LOVE) as the between-subject factor. Main effects of Time and Group, as well as Time × Group interactions, are reported. Within-group pairwise comparisons are presented descriptively where relevant

**Fig. 5:**
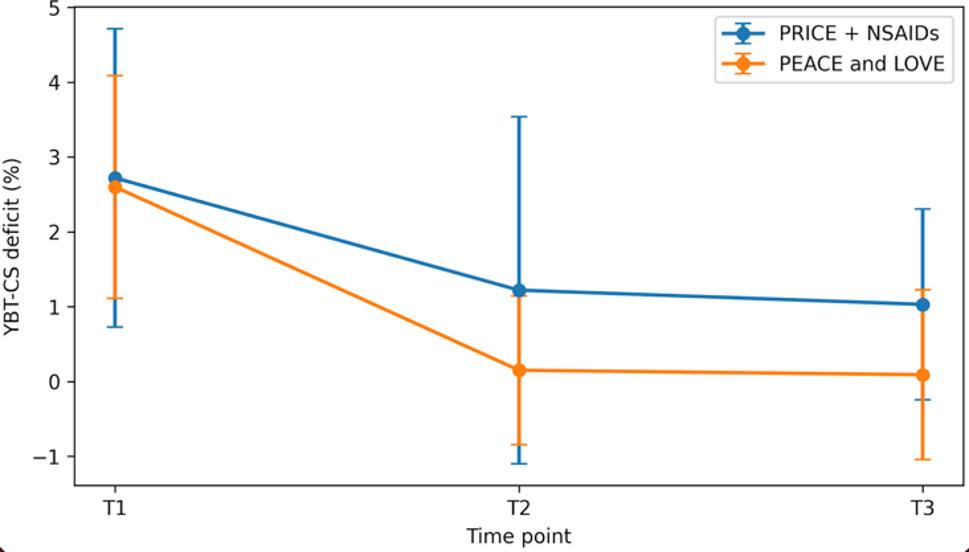
Dynamic balance (YBT CS deficit, %) (Mean ±95%, CI)

Mixed repeated-measures ANOVA demonstrated significant main effects of Time for IN strength at 60°/s and 120°/s, EV strength at 120°/s, ankle ROM at 60°/s and 120°/s, and YBT CS, indicating progressive recovery across the follow-up period. No significant main effects of Group and no Group × Time interactions were observed for any outcome (all p > 0.05), indicating similar patterns of change over time between the PRICE + NSAIDs and PEACE and LOVE groups. The longitudinal trends for strength, ROM, and dynamic balance outcomes are illustrated in Figs. 2, 3, 4 and 5, demonstrating near-linear improvement over time with overlapping 95% confidence intervals between groups.

## Discussion

This randomized study compared functional recovery following acute LAS in adolescents treated with either PRICE + NSAIDs or the PEACE and LOVE framework using objective biomechanical outcomes. Significant improvements over time were observed for strength, range of motion, and dynamic balance in both groups. However, no statistically significant group effects or Group × Time interactions were detected, indicating similar recovery trajectories between rehabilitation approaches during the 12–15-week follow-up period. Temporary plaster cast immobilization did not demonstrate a consistent functional advantage. Overall, these findings suggest that both management strategies are comparably associated with short-term functional recovery in this population.

### Comparison with up-to-date evidence

An umbrella review (24 systematic reviews) concluded that functional treatment surpasses immobilization for return to activity, swelling, and stiffness and also exercise therapy lowers re-injury risk [19]. The 2021 JOSPT Clinical Practice Guideline also recommends early optimal loading and balance exercises, emphasizing ROM and dynamic balance as main goals [20]. On the contrary, a 2025 meta-analysis reported no significant difference between functional treatment and immobilization considering pain level and physical function, which was evaluated over questionnaires, but groups were non-homogeneous [21]. Our findings are consistent with literature indicating that functional rehabilitation is at least comparable to immobilization in terms of short-term outcomes. In line with the 2025 meta-analysis reporting no significant differences between functional treatment and immobilization for pain and physical function [21], our data did not demonstrate a consistent advantage of temporary plaster cast immobilization for strength, ROM, or dynamic balance. Both rehabilitation protocols were associated with progressive recovery across follow-up. Although no significant Group × Time interactions were detected, descriptive within-group analyses indicated that some functional measures reached statistical significance earlier in the PEACE and LOVE group (IN strength at 60°/s; ROM at 60°/s and 120°/s; YBT CS). Because these patterns were not supported by significant interaction effects, they should be interpreted cautiously and considered hypothesis-generating rather than confirmatory. Larger, multicenter studies are needed to determine whether such trends represent clinically meaningful differences.

### Biological and functional rationale

The theoretical rationale underlying the PEACE and LOVE framework emphasizes early mechanical loading, patient education, and progressive physical activity, which are proposed to support collagen alignment, angiogenesis, and mechanotransduction. Evidence based data since 2019 supports prescribing balance and proprioceptive training to minimize injury rate [19, 20]. However, in the present study no statistically significant differences were detected between rehabilitation protocols. Therefore, while the biological mechanisms associated with early loading are supported by experimental and clinical literature, their direct contribution to differential outcomes in this cohort cannot be confirmed. Additionally, inflammatory biomarkers were not measured, and these physiological interpretations remain theoretical. Psychological components, such as optimism and self-esteem, can set realistic goals of rehabilitation and may lower medication intake, which are included into the PEACE and LOVE framework [20]. However, such effects were not directly evaluated in this study.

### Importance of avoiding anti-inflammatory modalities and cold therapy

Emerging literature suggests that anti-inflammatory medications and excessive icing may interfere natural healing by hindering macrophage activity and revascularization [7–9]. In the present study, no statistically significant differences in functional recovery were observed between participants managed with NSAIDs and icing and those treated under the PEACE and LOVE framework. Therefore, our findings do not demonstrate a functional advantage of routine anti-inflammatory or cryotherapy use in the short term. However, this study was not designed to directly evaluate inflammatory processes or long-term tissue healing outcomes. However, the avoidance of NSAIDs should be approached carefully, especially in children, where short-term use for pain control is relatively safe [22]. While emerging evidence suggests anti-inflammatory strategies for tissue healing support, NSAIDs remain appropriate for acute pain control when necessary [23]. Further research evaluating short- and long-term safety and functional outcomes of reduced NSAIDs use would be valuable.

### Dynamic balance and proprioception assessment

The YBT showed its sensitivity to group differences and our study results adds up with literature verifying its reliability for lower-extremity functional assessment [24]. Significant improvements in dynamic balance were observed across the follow-up period in both rehabilitation groups. No statistically significant Group or Group × Time interaction effects were detected, indicating comparable short-term balance recovery under both protocols. Although descriptive within-group analyses suggested that some balance-related changes reached statistical significance earlier in the PEACE and LOVE group, these patterns were not supported by interaction effects and should therefore be interpreted cautiously. As CAI and reinjury were not assessed, no conclusions regarding long-term preventive effects can be drawn.

### Immobilization and rehabilitation models

Our research data once again show no significant benefit from plaster cast immobilization, supporting contemporary evidence that functional splinting is equivalent or superior to immobilization for LAS recovery [19, 21]. Exploratory analyses demonstrated significant recovery over time in both cast immobilized and non-immobilized subgroups; however, no consistent main effects of cast immobilization or sustained time × group interactions were observed across strength, ROM, or dynamic balance outcomes.

Although isolated early differences in EV strength were observed at specific time points in the cast-applied subgroup, these findings were not persistent and were not accompanied by parallel improvements in ROM or balance. Descriptive within-group analyses indicated that certain balance and ROM measures reached statistical significance earlier in the non-immobilized subgroup; however, in the absence of significant between-group or interaction effects, these findings should be interpreted as exploratory. Such variations likely reflect early variability related to pain, apprehension, or voluntary muscle activation rather than a true physiological effect of plaster casting [25].

Overall, temporary immobilization did not demonstrate a consistent or clinically meaningful advantage in neuromuscular recovery. Given the rapid neuromuscular adaptability of adolescents, rehabilitation strategies emphasizing early functional loading with splint support appear preferable to prolonged immobilization following LAS.

### Additional measures and future perspective

Recent studies show that manual therapy combined with exercise can improve results in foot dorsiflexion ROM and pain control [26–28]. Such interventions could be incorporated into structured rehabilitation programs, including those emphasizing early loading and neuromuscular training. Future research should evaluate long-term re-injury rates, return-to-sport timelines, and patient-reported outcomes, which were not captured in this study.

### Clinical significance

The PEACE and LOVE framework may represent a biologically informed and patient-friendly approach to LAS management in adolescents, emphasizing education, early optimal loading, and functional rehabilitation. Despite being a more recently proposed rehabilitation framework, PEACE and LOVE demonstrated short-term functional outcomes comparable to those observed with the traditional PRICE + NSAIDs approach. In the present study, both rehabilitation protocols were associated with comparable short-term improvements in strength, ROM, and dynamic balance. No statistically significant between-group differences were detected.

The magnitude of these changes was modest, and their clinical relevance should be interpreted cautiously. Future studies incorporating minimal clinically important difference thresholds, patient-reported outcomes, and longer follow-up are needed to determine practical impact. Given the single-center design, application of these findings should be considered in the context of local clinical resources and expertise.

### Study limitations

This study has several limitations. Although outcome assessors were blinded, patients and the treating clinician were aware of treatment allocation due to nature of protocols, which may have introduced performance bias. The study was conducted at a single center in Lithuania, so generalizability to other clinical settings or populations may be limited. The sample size was adequate for primary outcomes but insufficient for subgroup comparisons, and the follow-up period was limited to 12–15 weeks, preventing assessment of re-injury rates or long-term functional recovery. Although recurrence is one of the main clinical concerns following LAS, this study did not evaluate ankle sprain recurrence as an outcome, as recurrent sprains often occur after full return to sport participation over a longer period. Although three re-injuries occurred during the study period (1 in the PRICE + NSAIDs group and 2 in the PEACE and LOVE group), all cases were attributable to non-compliance with the treatment protocol, specifically premature return to sports activities without wearing the prescribed lace-up ankle splint. Therefore, these events were considered adherence-related rather than reflective of protocol effectiveness. Longer-term follow-up is required to determine whether the functional improvements observed translate into reduced recurrence and CAI risk. Furthermore, an important limitation of this study is that we did not assess weight-bearing dorsiflexion (DF) ROM specifically, which is known to influence dynamic balance performance, particularly in the anterior reach of the YBT. Although we measured ankle ROM via isokinetic testing, this does not fully capture functional DF mobility under weight-bearing conditions. Previous work has shown that weight-bearing DF ROM is used in studies evaluating dynamic tasks and landing stability [29]. Additionally, separate research has demonstrated that DF ROM is correlated with YBT anterior reach outcomes in athletic populations [30]. Therefore, the absence of a specific DF ROM measure may have influenced our dynamic balance results, and future work should include standardized weight-bearing DF assessments to clarify its contribution. Furthermore, patient-reported outcomes (e.g., pain, perceived instability, return-to-sport readiness) were not collected, which restricts interpretation of clinical relevance beyond objective biomechanical measures. Finally, adherence to home-based exercise in the PEACE and LOVE group and to NSAID/cryotherapy use in the PRICE group relied on self-report, and compliance could not be objectively verified. Future studies should incorporate structured adherence tracking such as digital exercise logs, wearable motion data, or supervised session counts.

## Conclusions

Both rehabilitation protocols were associated with progressive functional recovery following acute LAS in adolescents. No statistically significant between-group differences or differential recovery trajectories were detected over 12–15 weeks. These findings suggest that an active, education-focused rehabilitation approach yields short-term functional outcomes comparable to traditional PRICE + NSAIDs management. Further research with larger samples and longer follow-up is required to determine long-term clinical relevance.

## Supplementary Information

Supplementary Material 1.

Supplementary Material 2.

## Data Availability

The datasets generated and analyzed during the current study are not publicly available due to privacy reasons, but they are available from the corresponding author on reasonable request.

## Abbreviations

A: Group A (PRICE + NSAIDs treatment group)
ATFL: Anterior Talofibular Ligament
B: Group B (PEACE and LOVE treatment group)
bpm: Beats per minute
CAI: Chronic Ankle Instability
CE: Conformité Européenne (product safety certification)
CFL: Calcaneofibular Ligament
CS: Composite Score (Y-Balance Test)
DF: Dorsiflexion
ED: Emergency Department
EV: Eversion
ICC: Intraclass Correlation Coefficient
IQR: Interquartile Range
IN: Inversion
JOSPT: Journal of Orthopaedic & Sports Physical Therapy
LAS: Lateral Ankle Sprain
LUHS: Lithuanian University of Health Sciences
NSAIDs: Non-Steroidal Anti-Inflammatory Drugs
PEACE and LOVE: Protection, Elevation, Avoid anti-inflammatories, Compression, Education + Load, Optimism, Vascularization, Exercise
PRICE: Protection, Rest, Ice, Compression, Elevation
PTFL: Posterior Talofibular Ligament
ROM: Range of Motion
ROAST: Rehabilitation of Acute Sprain Taskforce (International Ankle Consortium)
rpm: Revolutions per minute
SD: Standard Deviation
SPSS: Statistical Package for the Social Sciences
TQ/BW: Peak Torque normalized to Body Weight
