# Supplementary material for: PRICE (Protection, Rest, Ice, Compression, Elevation) vs. PEACE and LOVE (Protection, Elevation, Avoid anti-inflammatories, Compression, Education and Load, Optimism, Vascularization, Exercise) in adolescent lateral ankle sprain rehabilitation: a randomized prospective comparative study of muscle strength and dynamic balance
Source: BMC Sports Sci Med Rehabil. 2026 Mar 17;18:213. doi: 10.1186/s13102-026-01651-7 (PMC13107599; doi:10.1186/s13102-026-01651-7)
Supplement: Supplementary file 2 — Supplementary Material 2. [file 13102_2026_1651_MOESM2_ESM.docx]

Appendix A. Rehabilitation programs

*A.1. Initial Management for Both Groups*

All patients were initially evaluated and treated in the Pediatric ED of LUHS Kaunas Clinics, with subsequent follow-up in the outpatient LUHS clinic. When immobilization was applied in the emergency setting, it consisted of a temporary plaster cast based on clinical symptoms, which was removed at the first outpatient visit, 1-4 days after injury; thereafter, all participants were fitted with a lace-up ankle splint. Crutches were provided during the first days to support activity protection in cases of pain or antalgic gait. Functional assessments were conducted at three standardized time points: 1–2 weeks, 5–7 weeks, and 12–15 weeks after injury. Exercise instruction and progression were delivered by treating the orthopedic surgeon, while outcome assessors remained blinded to group allocation.

****Appendix A Table 1.** Group A and Group B treatment protocols.**

| **Rehabilitation Phase** | **PRICE + NSAIDs (Group A)** | **PEACE and LOVE (Group B)** |
| --- | --- | --- |
| Acute Phase (Weeks 1–2) | Protection with lace-up ankle splint; Rest and avoidance of weight-bearing for 3–5 days depending on pain; Ice application 4× daily for 15–20 minutes for first 2 weeks; Compression and elevation; Ibuprofen 3×/day according to body weight. No structured rehabilitation exercises were introduced during this phase. Gradual weight bearing return depending on pain recommended. | Protection with lace-up ankle splint, elevation, compression; Avoid NSAIDs and icing; Paracetamol for pain; Rest and avoid weight-bearing for 3-5 days, then gradual return; Education on healing process. Home exercise programme: • Elastic band strengthening plantar flexion/dorsal flexion/inversion/eversion: 4×10 slow repetitions daily (concentric + eccentric) • Balance: standing on a rolled towel 1 min, 2× daily, Exercises done with both legs. Exercises performed strictly pain‑free. |
| Subacute Phase (Weeks 3–6) | Return to full weight-bearing in lace-up splint; Strengthening and impact exercises not recommended; Oedema managed with elevation and icing; NSAIDs continued if needed. | Progressive loading as tolerated; Active ROM and elastic resistance strengthening continued; Balance training advanced to single‑leg stance on balance cushion/BOSU ball; Aerobic training (cycling/swimming). Warm–neutral contrast therapy (36–40°C to 20–25°C, 1–3 cycles, 1–2 min each), no cold immersion. Progression rule: pain ≤3/10 VAS and no next‑day swelling increase. |
| Rehabilitation Phase (Weeks 7–12) | Lace-up splint not required for daily living; Gradual activity increase; Dynamic sports avoided; Splint recommended if light running begins. | Progressive resistance and neuromuscular training; Moderate aerobic exercise; Plyometric and balance drills added; Sport-specific drills after Week 10 under supervision. |
| Late Phase (Weeks 12–15) | Return-to-sport allowed when: no pain, full ROM, ≥90% limb symmetry index. | Same return-to-sport criteria; Emphasis on self-management and re‑injury prevention. |

**Appendix B. Exploratory analysis of temporary plaster cast immobilization**

***B.1 Effect of cast immobilization on functional outcomes***

Functional outcomes were additionally explored according to the use of temporary plaster cast immobilization during the acute phase. Functional outcomes between these groups are summarized in **Appendix B Table 1.** Mixed repeated-measures analysis of variance demonstrated **significant main effects of time** for ROM at both 60°/s and 120°/s, indicating progressive recovery across the three testing sessions (1–2 weeks, 5–7 weeks, and 12–15 weeks) in both cast-applied and non-cast subgroups. **No significant main effects of immobilization group** and **no time × group interactions** were observed for ROM at either angular velocity, suggesting that plaster cast immobilization did not influence the overall trajectory of ROM recovery.

For peak torque normalized to body weight (Peak TQ/BW), significant main effects of time were observed for both IN and EV strength at 60°/s and 120°/s. However, **between-group effects and time × group interactions were generally not significant**, indicating comparable strength recovery patterns irrespective of cast use. An exception was observed for EV Peak TQ/BW at 60°/s and at 120°/s, where a **significant time × group interaction** was detected. Within-group comparisons demonstrated a significant improvement from T1 to T3 in the cast-applied subgroup, whereas the corresponding change in the non-cast subgroup did not reach statistical significance. Despite this interaction, no consistent or sustained between-group differences were observed across other strength outcomes or time points.

Dynamic balance, assessed using the YBT CS, showed a **significant main effect of time**, reflecting overall improvement across follow-up. No significant group effects or time × group interactions were detected. Within-group analyses indicated a significant improvement between T1 and T2, as well as between T1 and T3, in the non-cast subgroup, whereas changes in the cast-applied subgroup did not reach statistical significance. These findings suggest earlier balance recovery in participants without plaster cast immobilization, although the overall recovery pattern did not differ statistically between groups.

Overall, exploratory ANOVA-based analyses indicate that **temporary plaster cast immobilization did not confer a consistent functional advantage** in strength, ROM, or dynamic balance recovery. Observed subgroup-specific improvements at isolated velocities or time points should be interpreted cautiously, as they were not accompanied by robust group effects or consistent interaction patterns.

**Appendix B Table 1.** Comparison of functional recovery between participants who received temporary plaster cast immobilization during the acute phase and those who did not. Values are presented as **mean (SD)** and expressed as side-to-side deficits calculated as the difference between the uninjured and injured limb (U − I). Functional outcomes were assessed at three time points: **T1 (1–2 weeks post-injury), T2 (5–7 weeks), and T3 (12–15 weeks).** Isokinetic testing was performed at **60°/s and 120°/s** for inversion (IN) and eversion (EV) peak torque normalized to body weight (Peak TQ/BW) and range of motion (ROM). Dynamic balance was assessed using the **Y-Balance Test composite score (YBT-CS).** Statistical analysis was conducted using **mixed repeated-measures analysis of variance (ANOVA)** with **Time (T1–T3)** as the within-subject factor and **Immobilization (cast applied vs not applied)** as the between-subject factor. **Greenhouse–Geisser correction** was applied when the assumption of sphericity was violated. Main effects of Time and Immobilization, as well as Time × Immobilization interactions, are reported. **Within-group pairwise comparisons are presented descriptively where relevant.** Abbreviations: IN – inversion; EV – eversion; Peak TQ/BW – peak torque normalized to body weight; ROM – range of motion; YBT-CS – Y-Balance Test composite score; U – uninjured limb; I – injured limb.

| Variable | Time | Group | | Time effect | | | Group effect | | | Time × Group | | |
| --- | --- | --- | --- | --- | --- | --- | --- | --- | --- | --- | --- | --- |
|  |  | Cast applied | Cast not applied | df | F | p | df | F | p | df | F | p |
| Peak TQ/BW IN (U–I) % at 60°/s | T1 | 3.59 (7.95) | 1.84 (7.79) |  |  |  |  |  |  |  |  |  |
|  | T2 | 2.08 (10.27) | 0.32 (7.50) | 1.78, 111.84 | 6.40 | 0.003 | 1, 63 | 0.08 | 0.777 | 1.78, 111.84 | 2.14 | 0.128 |
|  | T3 | −1.86 (11.47) | 0.03 (8.63) |  |  |  |  |  |  |  |  |  |
| Peak TQ/BW EV (U–I) % at 60°/s | T1 | 3.37 (4.72) | 1.38 (6.91) |  |  |  |  |  |  |  |  |  |
|  | T2 | 2.13 (6.17) | 2.75 (6.35) | 1.95, 122.73 | 3.46 | 0.036 | 1, 63 | 0.00 | 0.951 | 1.95, 122.73 | 3.26 | 0.043 |
|  | T3 | −0.05 (5.52) | 1.56 (7.20) |  |  |  |  |  |  |  |  |  |
| ROM (U–I) ° at 60°/s | T1 | 4.77 (11.56) | 2.82 (11.56) |  |  |  |  |  |  |  |  |  |
|  | T2 | −0.31 (11.94) | −1.40 (9.96) | 2, 126 | 13.10 | <0.001 | 1, 63 | 0.15 | 0.704 | 2, 126 | 0.46 | 0.631 |
|  | T3 | −3.47 (8.65) | −2.81 (9.17) |  |  |  |  |  |  |  |  |  |
| Peak TQ/BW IN (U–I) % at 120°/s | T1 | 4.28 (8.20) | 3.23 (6.12) |  |  |  |  |  |  |  |  |  |
|  | T2 | 2.59 (8.02) | 1.33 (5.53) | 1.61, 99.97 | 10.26 | <0.001 | 1, 62 | 0.28 | 0.596 | 1.61, 99.97 | 0.30 | 0.692 |
|  | T3 | −0.29 (7.75) | −0.26 (6.53) |  |  |  |  |  |  |  |  |  |
| Peak TQ/BW EV (U–I) % at 120°/s | T1 | 4.49 (5.70) | 1.88 (4.22) |  |  |  |  |  |  |  |  |  |
|  | T2 | 0.73 (5.14) | 1.42 (5.44) | 1.77, 109.94 | 8.07 | 0.001 | 1, 62 | 0.42 | 0.518 | 1.77, 109.94 | 3.72 | 0.032 |
|  | T3 | 1.00 (4.56) | 0.85 (5.58) |  |  |  |  |  |  |  |  |  |
| ROM (U–I) ° at 120°/s | T1 | 4.34 (11.48) | 3.41 (11.71) |  |  |  |  |  |  |  |  |  |
|  | T2 | 0.68 (11.91) | −2.13 (9.75) | 1.87, 116.04 | 13.28 | <0.001 | 1, 62 | 0.23 | 0.635 | 1.87, 116.04 | 0.78 | 0.455 |
|  | T3 | −3.79 (9.37) | −3.04 (9.28) |  |  |  |  |  |  |  |  |  |
| YBT CS (U–I) % | T1 | 1.96 (5.46) | 3.25 (4.28) |  |  |  |  |  |  |  |  |  |
|  | T2 | 1.31 (3.86) | 0.13 (5.69) | 1.82, 114.80 | 6.66 | 0.003 | 1, 63 | 0.02 | 0.884 | 1.82, 114.80 | 1.96 | 0.150 |
|  | T3 | 0.42 (3.31) | 0.67 (3.47) |  |  |  |  |  |  |  |  |  |

The longitudinal trends for strength, ROM, and dynamic balance outcomes according to plaster cast immobilization are illustrated in Appendix B Figures 1–4, showing progressive recovery over time with overlapping 95% confidence intervals between subgroups.

**
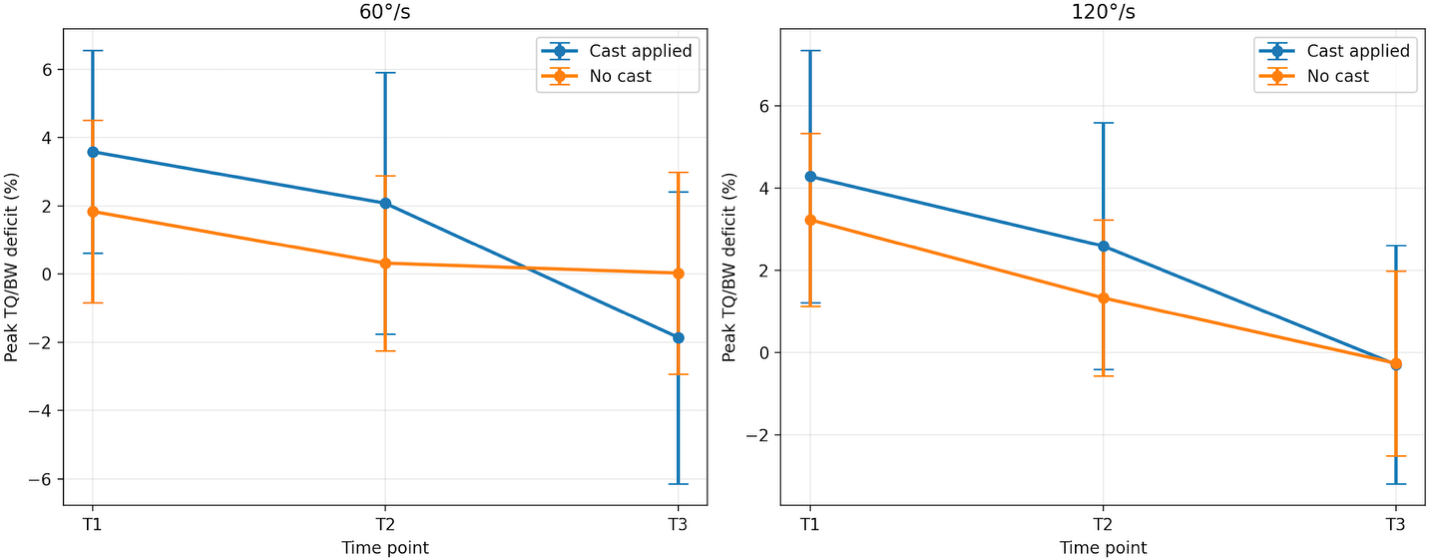
Appendix B** **Figure 1.** Inversion strength (Peak TQ/BW deficit, %) (Mean ±95%, CI)


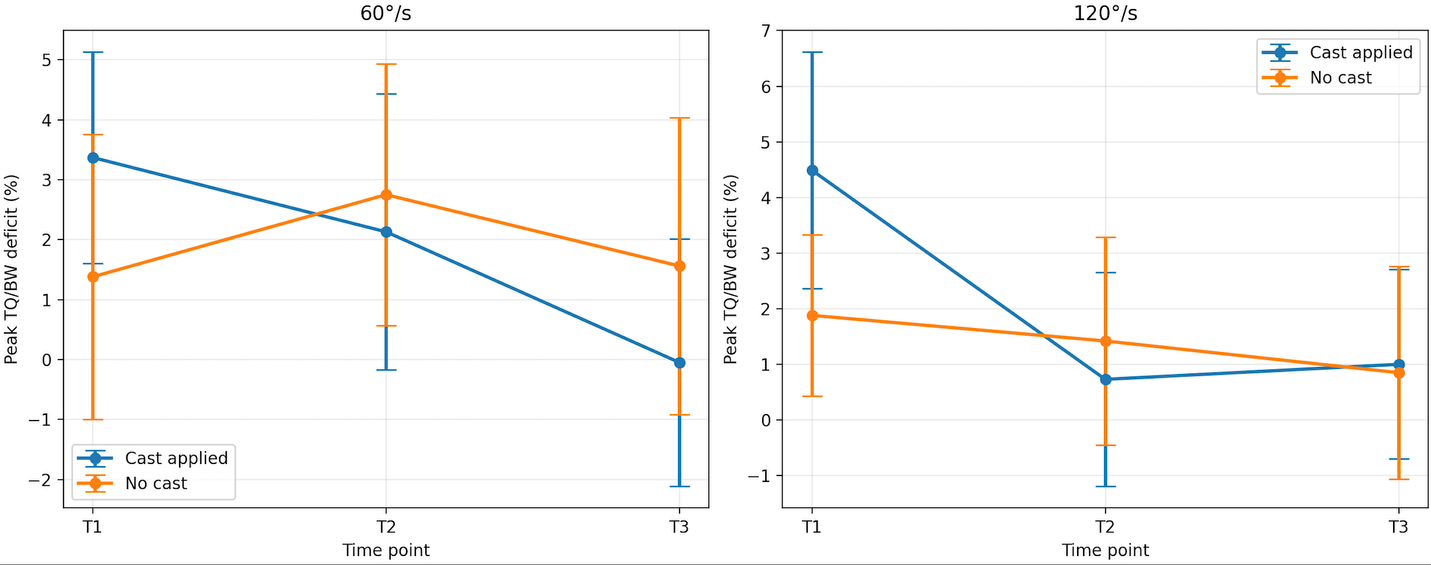


Appendix B Figure 2. Eversion strength (Peak TQ/BW deficit, %) (Mean ±95%, CI)


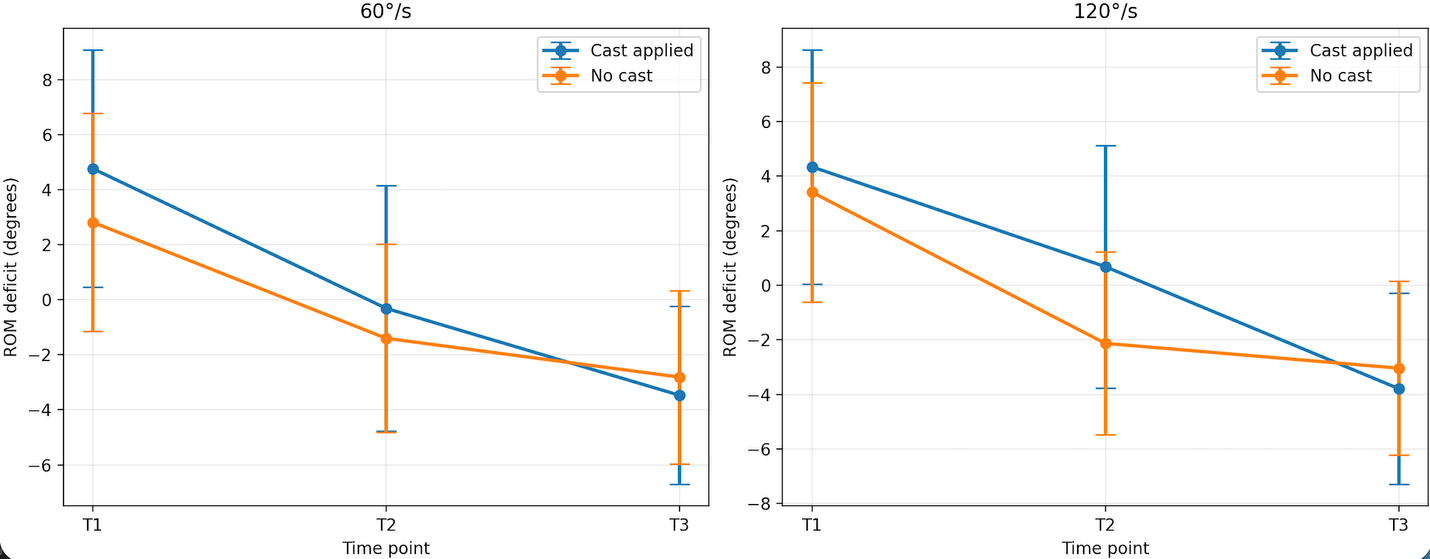


Appendix B Figure 3. ROM deficit (degrees) (Mean ±95%, CI)


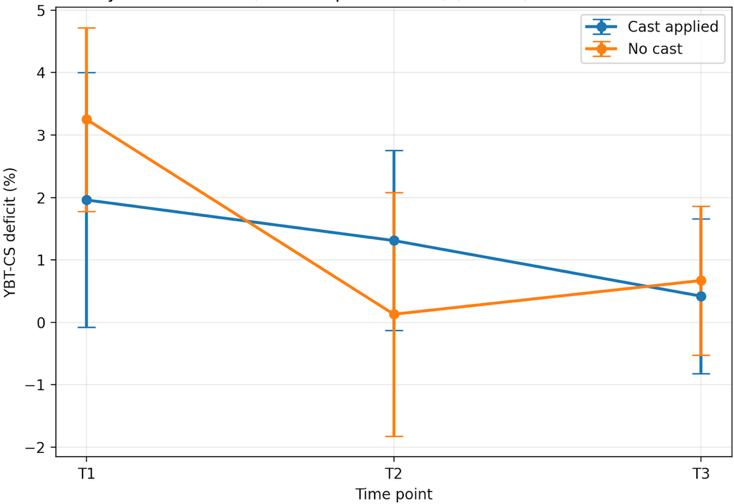


Appendix B Figure 4. Dynamic balance (YBT CS deficit, %) (Mean ±95%, CI)
